# Retrospect and Outlook of Research on Regional Haze Pollution in China: A Systematic Literature Review

**DOI:** 10.3390/ijerph182111495

**Published:** 2021-11-01

**Authors:** Li Li, Peng Deng, Jun Wang, Zixuan Wang, Junwei Sun

**Affiliations:** 1 School of Economics and Management, Harbin Institute of Technology, Shenzhen 518055, China; Liszsz@hit.edu.cn (L.L.); wzx_hitsz@foxmail.com (Z.W.); sunjunwei1002@hotmail.com (J.S.); 2 School of Business, Hunan University of Science and Technology, Xiangtan 411201, China; wangjunhnust@126.com

**Keywords:** haze pollution, systematic review, multidisciplinary, theoretical and empirical analysis, prospect outlook

## Abstract

Regional haze pollution, a severe atmospheric environmental problem, has profoundly harmful effects on the ecological environment, public health and the quality of economic development, and has accordingly attracted considerable attention from policymakers, researchers and the public. This article comprises a systematic literature review of the existing research on the theoretical mechanism, empirical analysis and institutional arrangement of regional haze pollution. As a result, it is found that various studies from multiple disciplines have touched upon the relevance of haze issues, including theoretical and experimental research on its formation, evolution and mechanisms from the perspective of the natural sciences, as well as empirical analysis and policy research on governance strategies, effects and mechanisms from the perspective of the social sciences, yet a systematic review and critical assessment synthesizing the above research is urgently needed. Future directions and research prospects are highlighted, showing that it is necessary to supplement and improve the theory and practice concerning the identification, measurement and assessment of haze pollution, as well as regional controlling strategies and policy implementation assessments. In short, in this review, we have aimed to help integrate the theoretical and empirical consensus in multidisciplinary fields, thereby promoting the accurate analysis, fine management and the development of precise policies in regards to regional haze pollution.

## 1. Introduction

In the context of world economic globalization and regional economic integration, the processes of industrialization, urbanization and modernization have accelerated significantly in China and globally. However, energy consumption dominated by coal combustion, as well as increases in industrial waste and the use of motor vehicles, along with city planning and construction accompanied by rapid economic growth, have all contributed to ambient haze pollution, one of the most serious sources of air pollution in the world [1,2]. Serious haze pollution results in poor air quality, which causes an estimated 2.6 to 4.8 million premature deaths per year worldwide [3,4,5]. From the “London Great Smog of 1952” incident, which caused surprise all over the world, to the “Chinese PM_2.5_ beyond-index” event, which led to a heavy haze and caused an extreme sensation in China [6], haze pollution has accordingly attracted considerable attention from policymakers, researchers and the public in China and globally, as its strong destructiveness seriously endangers public safety, human health and well-being and quality of life [7], significantly reducing the quality of economic development [8].

**Figure 1:**
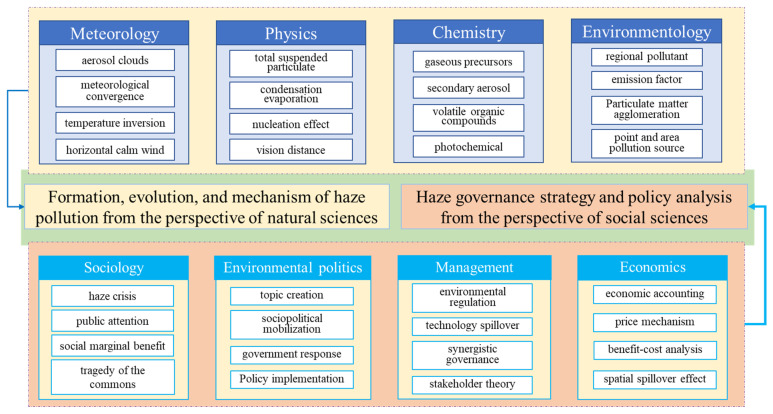
The structural framework and research focus of the existing literatures.

In a long-term development process, the formation, evolution and mechanisms of haze pollution have been studied and explored as a scientific problem, and the spatial correlations, government policy and social impact of regional haze have been followed and discussed as a social issue, and these issues continue to puzzles and trouble policymakers, researchers and the public. Many academic studies and publications have been presented and published in international authoritative journals and conferences, and several statistical methods, such as bibliometrics, knowledge graph methods and visualization software (Citespace), have been applied to quantitatively analyze the haze-related papers published in the Chinese academic journal database CNKI (China Knowledge Network) and the ISI-Thomson Reuters Scientific database (Web of Science). On this basis, the results show that foreign research on haze pollution appeared relatively early, mainly focusing on the causes, physical characteristics and driving mechanism of haze and policy assessments. In comparison, research by Chinese scholars on haze pollution has presented exponential growth since 2013, covering the main causes, scientific mechanisms, social impacts and governance strategies in regard to haze from the perspective of the natural sciences as well as the perspective of the social sciences. The core keywords have changed from “fog, smog, haze, particulate matter and aerosol” to “regional, health, economic impact, spatial autocorrelation”, and then evolved into “PM_2.5_, spatial spilling, decoupling effect, carbon emissions, and coordinated governance”. On the whole, the haze-related literature presents the characteristics of “high citation, high impact factor, and high growth”, and related studies and findings have been published in certain top journals such as the *American Economic Review* and *Management World*, and so on [9,10,11,12]. A large number of experts and scholars engaged in haze-related research have emerged from multidisciplinary fields, such as meteorology, physics, economics, and sociology. Certain Researches have made some progresses and many research findings have been made correspondingly. The existing literature can be basically divided into two major categories of studies, consisting of theoretical research on the formation, indicators and evolution and mechanisms of regional haze, as well as empirical research on its mechanisms of impact and policy analyses, from the perspectives of the natural sciences and the social sciences, respectively. The structural framework and research focus of the existing literature are illustrated in Figure 1.

In terms of attributes, mechanisms of formation, driving factors, social influences and governance strategies, related scholars have made certain breakthroughs and progress in their respective subdivisions, which have greatly enriched the theoretical, empirical and policy research results in regard to haze issues. However, several problems and challenges remain unresolved due to the complex attributes, spatial spilling and cross-regional characteristics of haze pollution, and the prevention and control of haze pollution has become a complex and diverse systems engineering problem [13]. As discussed in previous studies, the concept of haze pollution and its measurement indicators have not yet formed a unified and authoritative definition, so it is difficult to accurately identify local haze pollution and its different features, resulting in a lack of pertinence and effectiveness in haze pollution prevention and control policies. The formulation and implementation of relevant national policies requires precise identification and mastery of the components, attributes and stages of haze pollution types in different regions. Governance policies to combat cross-regional pollution issues have been analyzed and assessed mainly by means of cooperative game theory or non-cooperative game theory from the perspective of maximizing regional economic benefits. It is necessary to construct a dynamic model of the coordinated development of multi-regional economic systems and ecological systems, and to analyze the coordinated pathways of policies in regard to cross-regional haze pollution under the constraints of green development. Therefore, it is urgent to sort out and summarize the theories, methods and analysis frameworks from the different research perspectives, so as to provide suggestions and references for future theoretical studies and for the formulation and implementation of actual policies. On this topic, we found in the existing research that a small number of studies have been carried out, including macro-clustering analysis and the quantitative characterization of haze-related literature based on the “Scientometrics” method, but there is a lack of comprehensive excavation and analysis based simultaneously on micro, meso and macro perspectives in the representative literature [14].

**Figure 2:**
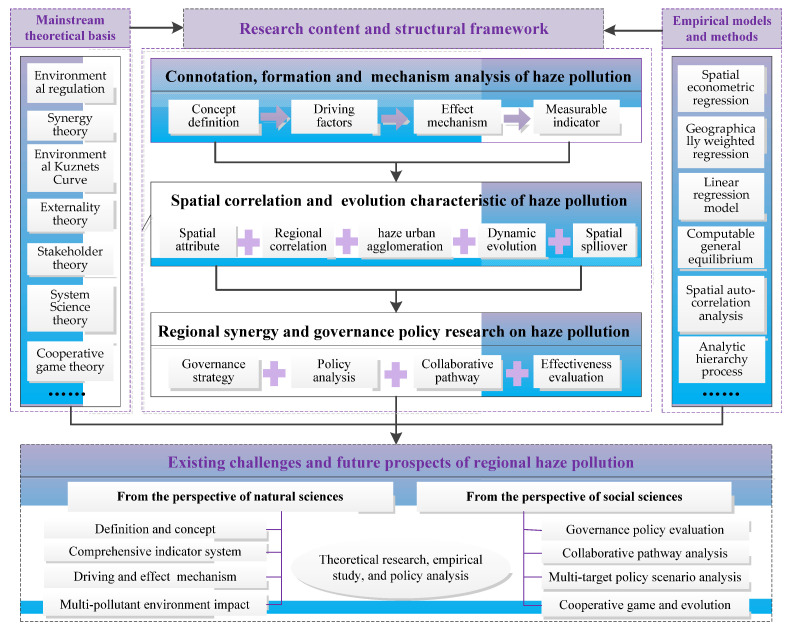
The overall framework of this review article.

On the above basis, in this article, we have summarized, analyzed and discussed future prospects in relation to the research content, development perspectives, theoretical frameworks and main viewpoints, selecting representative and groundbreaking studies from the existing theoretical, empirical and policy research literature. The overall framework of this article is illustrated in Figure 2. This study aimed to comprehensively summarize and comment on the status of the current research and existing problems, and open up new research perspectives and ideas for researchers and policymakers by tracking frontier hotspots and exploring future trends, which helps to provide theoretical references and methodological enlightenments for theoretical research and the formulation of policies in regard to cross-regional haze pollution in China.

The remainder of this article is arranged as follows. Section 2, Section 3 and Section 4, respectively, review and discuss the recent literature regarding the latest progress in studies of the formation, evolution and control of haze pollution in China. Furthermore, Section 5 summarizes the major progress in theoretical analytical frameworks and empirical quantitative methods. Section 6 presents a summary of major research findings, and current study limitations in existing studies, outlining topics that future studies should address. Finally, a future outlook on research prospects and a discussion of their policy implications are provided in Section 6.

## 2. The Connotations, Formation and Mechanism of Effects of Haze Pollution

**Figure 3:**
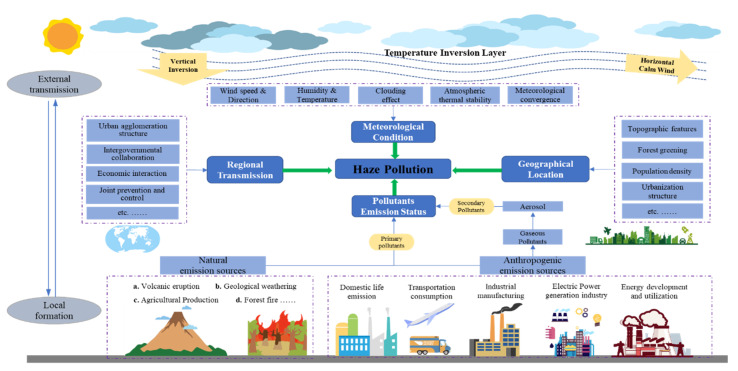
A scenario analysis of the mechanisms of the formation of regional haze pollution.

A large number of fruitful theoretical and experimental studies on the formation, evolution, and temporal and spatial characteristics of haze pollution have been carried out from the perspective of chemistry, environmental science and meteorology in recent years. Some progress has been made in terms of definitions, attributes and spatial characteristics; the composite components and internal and external causes of haze pollution have been clarified; and the spatial correlation characteristics of urban and regional haze have been summarized. The related studies, as the theoretical basis for an understanding of cross-regional haze pollution, are related to the practical effects of subsequent cross-regional prevention and control, governance, and policy research. As shown in Figure 3, a scenario analysis of the mechanisms of formation of regional haze pollution was constructed in this paper, based on the existing literature and the current research findings. This analysis indicated that the formation of regional haze pollution is caused together by special meteorological conditions, local pollutant emissions, geographic location characteristics and regional pollutant transmission, with the imbalance between the pollutant’s emission intensity and the atmospheric environment’s capacity leading to “meteorological convergence” and further causing extreme pollution conditions.

### 2.1. Basis Research on Concepts, Attributes and Evolution Characteristics

Haze pollution, as an interdisciplinary hot issue, has been widely discussed and studied by experts and scholars from different disciplines. However, the concept of haze has not yet been authoritatively defined, and the exact definition has always been controversial and relatively vague due to differences in research perspectives, methods and focuses. The indicator variables used to measure haze have developed continuously over time, and the evaluation indicators need to be further improved. Over the years of tackling difficulties, trends have gradually been recognized, characterized by the interdisciplinary and cross-border integration of haze pollution studies, progressing from continuous controversy to a general consensus.

#### 2.1.1. Concept Definition

The earliest studies on haze pollution mostly explained the concept of smog based on certain meteorological phenomena, including fog, smog and haze. Fog or smog is considered to be tiny water droplets formed by the condensation of water vapor in the air, whereas haze is regarded as fine dry particulate matter that is invisible to the eye in the air [15,16]. ‘Asian brown cloud’ was the earliest academic term used to describe the haze phenomenon in the world, denoting a brownish aerosol cloud layer with a thickness in the range of 3 km that has aroused the eager attention of academia. The “Ground Meteorological Observation Specifications” issued by the China Meteorological Administration in 1979 pointed out that this haze appears with a large number of fine dry dust particles floating uniformly in the atmosphere [17]. Since then, the researches on concepts, properties and causes of haze have been carried out in the fields of meteorology, physical chemistry and environmental science. Aerosols, meteorological convergence and pollutant transmission were discussed first—it is believed that haze is the phenomenon of reduced visibility caused by aerosols such as suspended solids and organic matter in the atmosphere [18,19]. Subsequently, the chemical mechanism and physical properties were studied, and it was found that haze is composed of a large number of fine dry particles in the air and that the accumulation of secondary aerosols often triggers serious haze pollution incidents [20,21]. Based on the analysis of on-site monitoring, indoor experiments and numerical simulation results, it was found that there are fine particles can be generally observed gathering in conditions with low visibility. Haze conditions and particulate pollution are only different descriptions of the same phenomenon. Haze pollution is a weather phenomenon based on visibility, and particulate pollution is based on the concentration levels of PM_2.5_ and PM_10_ particles [22,23]. In summary, haze pollution has been generally considered to be an extreme condition characterized by the deterioration of visibility near the ground caused by aerosol pollution due to the emission of air pollutants, including both the primary particulate matter directly emitted by the pollution source, and the secondary particulate matter formed by gaseous precursors (SO_2_, NO_2_, VOCs, etc.). The formation of haze is the result of the combined effect of special meteorological conditions, local pollutant emissions, characteristics of the geographic location and regional pollution transmission. The transformation and evolution of particulate matter is achieved by affecting the physical and chemical processes of the atmosphere [19,24,25].

#### 2.1.2. Attribute Characteristics

Haze pollution is generally considered to be the comprehensive result of the combined effects of multiple atmospheric pollutants. Its chemical composition and mechanism of formation are very complex, and the composition, hazards, and impact levels of haze pollution formed under different time and space conditions are different. Existing studies on the chemical composition and physical characteristics of haze have shown that particles of different sizes have different chemical compositions, and their sources and formation mechanisms are quite different, with particulate matter generally being distributed in four modes in the atmosphere, including “nucleation mode, Aitken mode, accumulation mode, and coarse mode”, and so on [25]. In addition, it has been pointed out that particulate matter is a complex mixture composed of inorganic components. The conditions have obvious differences in terms of the proportions of different regions and times, and the external and internal mixing forms are common in the ambient atmosphere [26]. In addition, based on the perspective of biomedicine, some scholars have found more than 1300 microorganisms through genetic sequencing and composition analysis of aerosol particles sampled in haze weather, and inferred that the frequent occurrence and expansion of haze in China is related to regional microbial populations and soils. Severe nonpoint-source pollution of water sources is closely related, which affects the speed of formation and diffusion, and the leap and sudden growth in the volume of condensation nodules [19]. The above studies indicate the complexity of the chemical composition and mechanism of the formation of haze pollution. A single pollutant indicator cannot fully reflect the basic nature and impact of haze pollution. Therefore, it is a key point and a research challenge to build the most representative measurement indicators and measurement variables for haze pollution.

### 2.2. Measurement Indicators and Evaluation

Based on the official statistical standards, data availability and data sources, three types of indicators are commonly used in empirical studies and policy analyses on haze pollution, including the concentration indicators based on ground-monitoring particulate matter concentrations of PM_2.5_, PM_10_ and total suspended particulate matter (TSP); the comprehensive air index indicators integrating SO_2_, CO_2_, NOx and other pollutants, such as air quality index (AQI) or air pollution index (API); and the satellite monitoring indicators, which sense and monitor optical thickness and mass concentrations with spectroradiometers (MODIS), atmospheric infrared detectors (AIRS) and other satellite remote sensing approaches. International attention to the prevention and control of atmospheric particulate matter began with “the Great Smog of 1952”. In terms of the criteria and measurement indicators for haze pollution, the black smoke and sulfur dioxide emitted by combustion were noticed initially, and then attention is paid to SO_2_, NO_2_, PM_10_ and API levels as air pollution increased in industrialized areas, and now it is mainly focused on PM_2.5_ and AQI indicators and inversion values of satellite remote sensing data and other indicators. Several studies have indicated that the range of influence of PM_2.5_ particles is mainly on the local scale, and can be extended to the regional scale or global scale, as haze pollutants are mainly composed of atmospheric particulate matter, mainly including PM_2.5_ and PM_10_ particles [23,27]. Different types of indicators such as concentration indicators, comprehensive index indicators or satellite monitoring indicators have been applied in empirical studies on economic impact, mechanisms of action and spatial characteristics, showing that China’s haze pollution generally has spatial spillover effects and regional agglomeration characteristics, that economic and social factors have a significant relationship with haze pollution, and that this is the main formation and driving factor [28,29,30,31,32,33,34]. Concentration data have the advantage of better continuity and availability, but are limited by the differences in monitoring technology, coverage and statistical caliber. Comprehensive index data can comprehensively reflect the air pollution status through the consideration of the discharge of multiple pollutants, but they are affected by the monitoring standards and statistical errors. Satellite monitoring data have the advantages of coverage, monitoring standards and data caliber, but they lacks the objectivity of on-site monitoring, and are affected by meteorological conditions, transmission processes, and data retrieval techniques. To sum up, the principles for selecting reference indicators include practicality, scientific standardization, comprehensiveness, relative independence and comparability. Furthermore, the measurement indicators of haze pollution should be combined with the concentration data from ground monitoring and remote sensing data, with the optimization of the two-stage spatial statistical model, and an index system suitable for different spatial scales and time evolution is critical. The comparative analysis and evaluation of the existing measurement indicator systems for haze pollution are shown below in Table 1 [20,21,26,35].

### 2.3. Driving Factors and Analysis of Mechanisms of Haze Pollution

The formation of haze is the result of the combined effect of special meteorological conditions, local pollutant emissions, geographic location characteristics and regional pollution transmission. The imbalance between the pollutant’s emission intensity and the atmospheric environment’s capacity lead to “meteorological convergence” and, furthermore, cause extreme pollution conditions. A large number of studies on the driving factors and dynamic mechanisms have been carried out, including local pollutant emissions caused by economic and social factors, special meteorological conditions caused by natural environmental factors, and cross-regional pollution transmission caused by the circulation and diffusion of air [36,37]. In the early stage, the environmental Kuznets curve (EKC) theory, a relatively mature theoretical analysis paradigm, was constructed by Grossman and Krueger, proposing that environmental pollution would present an inverted U-shaped trend that deteriorates first and then improves with economic growth [38]. Subsequently, a large number of theoretical and empirical studies have been carried out, but there have been constant disputes and controversy about EKC theory for a long time. At the same time, Tobler’s first law, an idea of spatial econometrics, has been put forward by Cliff and Ord and Waldo Tobler, and began to prevail in geography and regional economic research, and gradually expanded into the fields of environmental pollution and climate change [18,39]. The existing research on the effect and mechanism of economic and social factors on haze pollution has mostly relied on the improvement and expansion of the EKC model, the KAYA identical equation, the IPAT equation, the STIRPAT model and the ESDA model [28,40,41,42], the factors studied mainly include economic development level, industrial structure, urbanization, population agglomeration, foreign direct investment, energy consumption, transportation mode, fiscal decentralization, environmental governance, public concern and so on [43,44,45,46,47,48,49]. On this basis, it is necessary to combine the social development stage and economic growth mode of China in a specific period when discussing the impact of economic and social factors on the formation of haze pollution, because China’s industrialization and urbanization processes have gone through different stages. In terms of natural environmental factors, it has been found that features of the natural environment, such as temperature, humidity, pressure, wind speed and direction, clouding effects and meteorological convergence, and geographical location, even the accumulation and diffusion of atmospheric pollutants, have an important impact on the formation of haze pollution [20,22,50,51,52].

## 3. Spatial Characteristics and Dynamic Evolution of China’s Regional Haze Pollution

China’s haze pollution has significant spatial autocorrelation and spatial spillover effects. It shows a continuous spreading trend in space, and a gradually expanding range in terms of its distribution and transmission, which is mainly presented in urban agglomeration and metropolitan areas, where the economic growth is relatively developed and the agglomeration is significantly higher [29,53]. In this section, we aim to reveal the spatial correlation, dynamic evolution and regional collaborative pathway of China’s regional haze pollution with evidence from the representative urban agglomerations in China, by means of summarizing and analyzing the existing literature and policies, which could help to pave the way for the assessment and analysis of policy research on haze pollution prevention and control actions.

### 3.1. Evidence from the Beijing–Tianjin–Hebei Urban Agglomeration

The Beijing–Tianjin–Hebei urban agglomeration is a cluster region with the largest cross-regional area and the most extreme haze pollution in China. It has been proposed that a fragmented regional administration structure is the institutional cause that has made it difficult to eradicate haze pollution for a long time [54]. As presented in the existing literature, both environmental quality and economic benefits can be achieved at the same time. With the continuous improvements in haze governance and environmental quality, economic operations and industrial development have been upgraded and related environmental protection industries have grown constantly [55]. Certain empirical models and methods, such as the impulse response function, Markov region transformation model, spatial autoregressive model and exploratory spatial data analysis, are widely applied to studies on the Beijing–Tianjin–Hebei urban agglomeration [56]. Studies have indicated that the haze pollution has a zoning conversion effect, which causes effects between local and neighboring regions [57]. The pollution statuses of neighboring cities affect each other, and haze pollution in neighboring cities aggravates that of local cities [58]. Significant spatial agglomeration characteristics and spatial heterogeneity exist, and economic growth, urbanization level, industrial structure and population density, as well as technological level and opening-up level, are the main factors driving the aggravation of haze pollution [59,60].

### 3.2. Evidence from the Chengdu–Chongqing City Group

Some scholars have analyzed the spatial characteristics and control policies and point out that the coordination mode of haze pollution governance is a “task-driven” model, and that the constitutional framework, governance structure, operating mechanism and intergovernmental games affect the overall effect of cross-regional governance polices. The Chengdu–Chongqing City Group is the one of the most serious areas discussed in the literature, with continuous haze pollution and excessive particulate matter, which is affected by local and surrounding pollution sources. The “Air Pollution Joint Prevention and Control Agreement in Chengdu–Chongqing area” was signed and implemented in 2016. Certain empirical models and methods such as geographic weighted regression, the GIS spatial analysis method, the spatial Durbin model and social network analysis, have been applied in the existing studies [61,62]. It was found that haze pollution has a significant spatial correlation, and that the spillover effect of neighboring cities is obvious, but there is a time lag effect, so regional joint prevention and control is the top priority for pollution control and haze reduction [63]. The spatial correlation of PM_2.5_ particles is the main cause of the haze, which presents a multi-threaded complex network structure with strong connections, strong stability and obvious characteristics between cities. The PM_2.5_ measurements in the Chengdu–Chongqing urban agglomeration exhibit spatial dependence and spatial agglomeration, including differences in their temporal-spatial distribution, influencing factors and duration, compared with other regions such as the Beijing–Tianjin–Hebei urban agglomeration and the Pearl River Delta urban agglomeration. Policy recommendations include implementing special actions, optimizing industrial structures, controlling motor vehicle emissions, regional joint prevention and governance measures, and building urban PM_2.5_ information technology management platforms [64,65].

### 3.3. Evidence from the Pearl River Delta Urban Agglomeration

The Pearl River Delta urban agglomeration, as one of the most prosperous urban agglomerations in China, is the largest industrial production area, which cause a large amount of air emissions and haze pollution. Significant progress has been made in cross-regional research and the policy effects in regard to haze pollution in recent years. The cities in the Pearl River Delta agglomeration, applied to China’s macro-health production function and spatial econometric model, have been empirically studied and researchers have found that the negative externalities and spillover effects of haze pollution will seriously affect the public health of residents, including the direct impact of local cities and the spillover effects of neighboring areas. They have also found that PM_2.5_ particles have a greater impact in terms of health damage than that of PM_10_, relatively. It has thus been recommended to break administrative boundaries and implement cross-regional environmental protection cooperation and policy coordination [66]. The spatial correlation and the impact of foreign direct investment on haze pollution have analyzed and researchers have demonstrated that there is a significant spatial autocorrelation of haze pollution in the Pearl River Delta agglomeration, that the “Pollution Paradise” hypothesis is incorrect, and that foreign direct investment can improve haze pollution status [28]. GIS spatial analysis and the spatial Durbin model have been applied to explain the temporal and spatial evolution characteristics and driving factors in relation to PM_2.5_, indicating that the PM_2.5_ concentrations in urban agglomerations are high in the east and low in the west (divided by Hu’s line), as the hot spots are concentrated in the east, and the cold spots are concentrated in the west. Population density, industrialization and energy consumption have exacerbated PM_2.5_ levels, and foreign direct investment and urbanization have improved PM_2.5_ levels in the Pearl River Delta urban agglomeration [67]. Air pollutant emission reductions and climate change can effectively promote the improvement of regional air quality, especially under abnormal meteorological conditions [54].

### 3.4. Evidence from the Yangtze River Delta Urban Agglomeration

The Yangtze River Delta urban agglomerations, as the largest and most dynamic metropolitan economic area in China, have led to a decline in environmental quality and a bottleneck restricting regional economic development, seriously affecting people’s health and standards of living. The evolution characteristics and influencing factors on the temporal and spatial patterns of haze pollution in the Yangtze River Delta are critical research issues. The warning and emergency response mechanisms for heavy air pollution incidents have been highlighted to weaken the impact of sudden haze pollution on residents’ health, life and production activities [10,68]. Empirical models and methods such as the spatial Durbin model, the differences-in-differences model, the Granger causality test and the evolutionary game model have been applied in the existing studies. It has been found that the haze pollution in this area has a significant positive spillover effect, that the haze pollution and economic growth show an inverse “U-shaped” curve relationship, and that the advancement of urbanization is the main driving factor that aggravates the degree of haze pollution. The strength of the spillover effect is related to the geographical location and industrial structure. Studies have recommended adjusting industrial policies and optimizing the urban structure to promote the efficient management of haze pollution. The impact of population development factors on haze is heterogeneous, manifested in the suppression of quality factors, the promotion of scale factors and structural factors [67,68,69]. Furthermore, the policy data on cooperative governance in relation to air pollution and water pollution in 30 cities in the Yangtze River Delta urban agglomeration were analyzed based on the explanatory model of institutional collective action, and the results indicated that there are obvious differences in the level of pollutant cooperation governance, cooperation methods and the cooperation network structures in different environmental fields, which is rooted in the asset specificity and performance measurability of different fields [70].

### 3.5. Comparative Analysis and Discussion

#### 3.5.1. Comparative Analysis

Based on the above results and the conclusions regarding the selected representative urban agglomerations in China, it has been demonstrated that the mechanisms of formation of regional haze pollution are caused together by special meteorological conditions, local pollutant emissions, geographic location characteristics and regional pollutant transmission. China’s haze pollution has significant spatial autocorrelation and spatial spillover characteristics, which are common and similar across the different urban agglomerations; meanwhile, there is also significant spatial heterogeneity, which shows certain differences, including in the temporal-spatial distribution, the main driving factors and the duration. Furthermore, regional haze pollution, as a common challenge for all the urban agglomerations in China, requires a multi-subject governance strategy, including government guidance, market mediation and public organization guidance, as implemented and certified in the Pearl River Delta urban agglomeration. Moreover, cross-regional government cooperation and collaborative governance is the most effective means of administrative environmental regulation at present, as confirmed by the Beijing–Tianjin–Hebei urban agglomeration. Furthermore, economical governance instruments, which play a role in marketing adjustments, are primarily concerned in areas including economic growth, the urbanization level, industrial structure and population density, as well as technological level and opening-up level. Finally, there are obvious differences in the level of cooperative governance, co-operation methods and cooperation network structures in relation to pollution.

#### 3.5.2. Summary and Discussion

Haze pollution has obviously complex components and cross-regional characteristics, as air pollutants are free flowing and exhibit spatial spillover. Several researches have focused on the spatial autocorrelation of haze pollution, spatial spillover effects and spatial correlation characteristics. According to “Tobler’s First Law of Geography”, most spatial data are spatially dependent and feature accumulation and spillover characteristics. Therefore, spatial econometric methods such as spatial regression models, geographically weighted regression models and spatial simultaneous equation models have been commonly used in the existing researches [7,71,72]. China’s haze pollution has a significant spatial spillover effect, which is manifested as the agglomeration of high-value haze pollution areas and higher pollution-level areas, whereas low-value haze pollution areas tend to agglomerate with lower-level areas. [73]. Zhang. et al. [74] analyzed the formation and transmission process of PM_2.5_ in Beijing based on gray correlation theory and pointed out that the generation and evolution of PM_2.5_ is a dynamic random process, and the transmission mechanism includes the local energy structure, as well as the influence of regional transmission and the local urbanization process [75]. Some scholars have found that haze pollution has significant diurnal and seasonal changes and interannual variations from the perspective of time evolution characteristics. Specifically, the interannual variation of haze concentrations has roughly six change stages, and the change trend is mainly affected by the processes of industrialization and urbanization, economic development stages and air environment policies. The seasonal changes are more serious in winter and spring than in summer and autumn due to meteorological conditions, climatic environment and central heating policies. Diurnal change characteristics show that the peak travel time leads to more serious levels in the morning and evening, which are mainly affected by mobile emission sources, vehicle exhaust and traffic operation conditions [76,77,78]. In summary, it can be verified that haze pollution has obvious compound and regional characteristics, that these compound characteristics are embodied in the source, cause, evolution and meteorological convergence processes; and the regional characteristics are embodied in the spatial autocorrelation and spatial spillover effects at the scale of urban agglomerations.

## 4. Evolution Process and Policy Analysis on Haze Pollution Prevention and Control

### 4.1. Evolution Process and Progress

**Figure 4:**
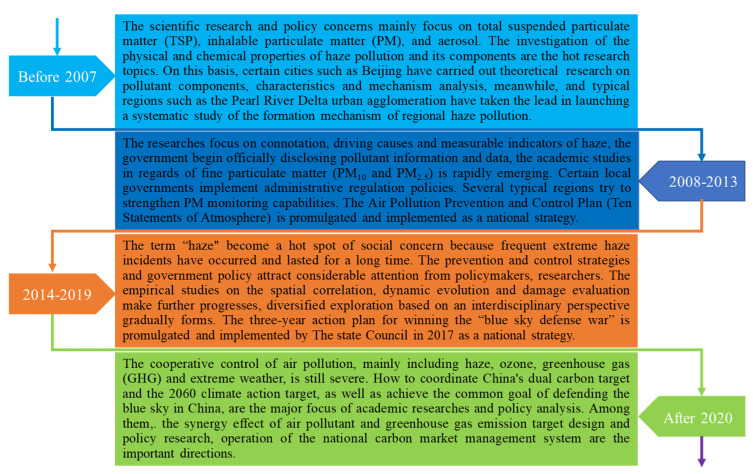
The evolution over time of research on China’s haze pollution.

In 2013, in an event known as the “severe haze incident in eastern China”, a number of severe haze pollution incidents occurred in China, and their frequency of occurrence, their spread and the severity of the pollution were unprecedented. Since then, the number of haze days in China has remained at high levels despite the numerous studies that have been conducted on this issue, the suggestions that have been proposed to solve the problem and the measures taken by the government to improve air quality [79,80]. According to the official data from the China Meteorological Administration, the severe haze incident in central and eastern China that broke out in early 2013 has affected the everyday lives of nearly half of China’ total population, covering about 2.5% of China’s land area across 25 provinces, and has set historical records for the number of extreme weather days. As a primary pollutant, the concentration value of fine particulate matter (PM_2.5_) has frequently exceeded 150 mg/m³, and has reached 300 to 500 mg/m³ in some typical areas, which far exceeds the limits of the World Health Organization’s safety standards and China’s air quality standards [81]. According to the “satellite haze map” taken by the National Aeronautics and Space Administration (NASA), a “grey Great Wall” from the north to the south enshrouds China, covering a land area of millions of kilometers. As a result, frequent extreme haze incidents have also awakened public opinion and brought attention to haze pollution. Taking the *People’s Daily* as an example, news reports related to haze have increased sharply. Certain consumption data showed that “the willingness to pay for haze governance” became the annual consumption keyword in 2013, and the sales of health protection and air purification industries doubled [82]. Based on the above, China is at an important stage of ambient air pollution control, and the unaddressed environmental risks and consequences at the regional and global levels pose major policy challenges. Air ollution, especially haze prevention and control, has been defined as a considerable livelihood issue and was upgraded to a national strategy by the Central Committee of the Communist Party of China. To solve the challenges arising from haze pollution in China, China’s government has implemented various renewable energy policies, emission reduction regulations and environmental standards. The Air Pollution Prevention and Control Plan (hereinafter referred as the “Ten Statements on the Atmosphere”), as a national policy and regulation similar to the “Clean Air” acts in the UK and USA, has been promulgated and implemented for the haze pollution. Since its implementation in 2013, air pollution governance policies have been optimized, urban air quality has been significantly ameliorated and cross-regional haze pollution has continued to improve. According to the “China Ecological Environmental Status Bulletin 2020” and “Air Quality Assessment Report Ⅶ by Peking University”, the average air quality in cities across the country has improved year by year, and the key indicators such as the number of haze pollution days, PM_2.5_ concentrations and the average number of days of heavy pollution, have dropped significantly. The evolution over time of haze pollution-related research in China is illustrated in Figure 4.

### 4.2. Governance Policy Analysis and Evalution

The scientific design and reasonable construction of haze-related policy is an important part of winning the battle against haze pollution. China’s government has issued multiple policies, regulations and plans to control haze pollution in the country. Several representative policy documents relating to China’s policies on haze pollution prevention and control are selected and analyzed in Table 2. It has been demonstrated that China’s policy system on haze pollution has been gradually established and steadily promoted, from the perspective of the time, level and regional distribution of the policy release. Several policy documents were released in two periods, in the years 2013–2014 and 2017–2018, which coincided with the time of the “Ten Statements on the Atmosphere” and “The Blue Sky Defense War”. Government departments at different levels have actively participated in policy formulation and implementation, and above all, industrial development and corporate transformation are important regulatory entities for the implementation of current policy, and there is a lack of restraint and regulation on residential consumption and public behaviors in current policies. The general policy in the early stage provided timely guidance for air pollution prevention and control, but special and targeted policies for haze pollution are still significantly insufficient and deficient. On the bases of the above, research on the policy expectations and the benefit evaluation of haze policies and regulations are considered to be vital.

#### 4.2.1. Short-Term Governance Policy Research

For short-term policy research on haze pollution, the effects of pollution source control and energy emission reduction policies on haze pollution are the main focus of research. Policy simulation methods such as regression discontinuity design, the synthetic control method, difference in differences and system dynamics have been adopted in existing studies. Studies demonstrate that some local governments use temporary measures to control air pollution and create “political blue sky” in certain periods, but this comes at the cost of retaliatory pollution and the air quality deteriorates again [83]. Several findings have been presented that the air pollution charging fee (APCF) policy has a shunting effect on traffic and an emission-reducing effect [10], which can alleviate traffic congestion to a certain extent, but its effect on haze pollution control is extremely limited [84], due to the acceleration of urbanization, industrial and energy structure, which cause rebound effects, inflection effects and agglomeration effects [85]. As a result, the policy tends to reduce the overall welfare of society and to increase haze pollution because it stimulates the purchase of a second vehicle, and the usage of older private cars is delayed [86]. In the study of energy conservation and emission reduction policies, it has been indicated that “coal-to-natural gas” policies can help to improve the quality of winter haze in northern China, as its cost is much higher than that of coal-fired supply, and clean coal is a future focus in studies of haze pollution control [40]. On the whole, the current relevant research conclusions demonstrate that short-term policies tend to have extremely limited effects because of the high social costs and welfare losses, and the temporary measures that create “political blue sky”, for example, tend to have rebound effects at the cost of retaliatory pollution.

#### 4.2.2. Long-Run Governance Policy Research

For long-term policy research on haze pollution, several studies have focused on administrative regulations and economic instruments which aim to improve the capabilities of the government, to optimize energy and industrial structures, to increase transportation emission efficiency and to transform economic development models. China’s air pollution control policies in the early years were categorized into three stages based on scientific evidence for the evolution of air pollution and the institutional background, whereas future studies are expected to reforming the governance scheme towards air quality improvement and health risk-oriented management [87]. From the perspective of specific policy measures, it has been pointed out that the key to controlling haze pollution is to strengthen the construction of new urbanization and to improve the utilization of public transport utilization [88], and the foundation lies in the optimization of the economic development structure and the adjustment of the industrial development structure [89]. Furthermore, it is critical to prioritize the development of public transportation, to optimize the road network and to improve clean-coal technology [90]. Reducing emissions is probably the most effective way to control haze pollution, but the effectiveness of its implementation varies depending on local economic and political circumstances. Furthermore, the policy effects of haze pollution are evaluated based on environmental carrying capacity, indicating that the “Ten Statements on the Atmosphere” policy measures have been active and effective; thus, more attention should be paid to reducing the frequency of heavy haze pollution, so as to control the sources of pollutant emissions in winter [91].

### 4.3. Research on Mechanisms for the Prevention and Control of Haze Pollution

It has been demonstrated that haze pollution depends not only on local emissions from a region, but also on transboundary emissions from neighboring regions, which requires regional cooperation across administrative areas and government departments to reduce pollutant emissions as well as to improve air quality [92]. In the process of policy-making, establishing joint prevention and control mechanism for regional haze pollution has become a consensus, due to the diffusion and spillover characteristics of haze pollution. As a result, synergy governance theory and inter-governmental cooperation theory are widely discussed to clarify the effects of mechanisms for the prevention and control of cross-regional pollutants [93,94]. Some scholars have analyzed the effects of mechanisms and the systems logic of collaborative governance. The dynamic mechanism of inter-governmental cooperation includes political mobilization mechanisms, systemic institutional mobilization mechanisms, and interest balance mechanisms of intergovernmental cooperation, and the driving force of collaborative governance comes from the combined effect of internal driving forces such as increasing expected income, external driving forces such as the promotion of public environmental protection awareness, and supporting forces such as government policy support [95,96,97]. More specific research findings have revealed that there is competition among local governments in relation to environmental regulation, which exists a comparable strategy of comparison rather than a differentiated strategy of dislocation competition [98]. Geographical location is also an important factor that affects enterprises’ pollution discharge decisions and the intensity of environmental regulations in various regions [99]. Extreme haze events in megacities are caused by the spread of by the expansion and spillover of surrounding cities, such as the Beijing–Tianjin–Hebei urban agglomeration, so joint prevention and control is a key policy [84]. It has been demonstrated that regional policy synergy has a significant positive effect on haze governance from the perspective of the quantified policy information, based on the gray relational method and the dynamic panel model. The degree of policy synergy in China is basically at the level between primary synergy and mild imbalance, with the slow evolution of synergy and large fluctuations. It is thus critical to strengthen regional policy synergy in order to improve the mechanism of synergistic governance in various regions [100]. The future haze pollution control measures should not only consider the coordinated control of regional air pollutants, but also the coordinated control of multiple air pollutants, as global warming is one of main reasons for the aggravation of haze pollution [101,102]. On the above basis, current problems and predicaments include local protectionism, race-to-the-bottom environmental regulation, as well as the lack of mature and effective policy synergy mechanisms, inter-governmental institutional systems.

### 4.4. Policy Research Overview on the Prevention and Control of Haze Pollution

Base on the analysis of short-term policies, long-term policies and mechanisms of the effects of haze pollution in China, taking into account the complexity of the driving causes, the natural agglomeration characteristics and the spatial spillover effect, governing strategies and policy implementations need to break the boundaries of administrative jurisdictions and establish cross-regional coordination mechanisms. This would play an effective role in establishing internal pollution compensation mechanisms, benefiting coordination mechanisms and “green GDP” competition mechanisms, realizing the sharing of environmental information and the unification of environmental regulatory actions because of the “leakage effect” and “free rider” behavior. It is critical to reassess the environmental governance effects of regional industrial transfer, and to formulate industrial transfer policies tailored to local conditions, as regional haze pollution is also significantly related to local industrial infrastructure and intergovernmental industrial policy. Moreover, this could realize the coordinated management of haze pollution with the joint participation of multiple subjects, including government environmental management and regulation, enterprise technological innovation and transformation and public implementation and feedback. From the perspective of the stakeholders of haze pollution, policy implications include seeking collaborative governance strategies for win–win cooperation and benefit sharing, specifically, introducing a public participation mechanism into the control of haze pollution, and disclosing environmental information and performance to the public.

## 5. Theoretical and Empirical Research Overview

Based on a systematic overview of the existing literature, in this section we analyze and summarize the current research progress and major findings on the theoretical analytical frameworks and empirical quantitative methods, as the formulation and implementation of policies require macroeconomic analyses and empirical demonstrations. As a result, the related studies, providing the theoretical basis and empirical support for cross-regional haze pollution control, are related to the practical effects of subsequent cross-regional prevention and control, governance and policy research.

### 5.1. Theoretical Basis and Analytical Framework

The formation of haze pollution is the result of several natural and social factors, specifically, the effects of special meteorological conditions, local pollutant emissions, geographic location characteristics and regional pollution transmission. Moreover, anthropogenic emissions and cross-regional transmission are closely related to economic and social activities. On this basis, economics researchers have gradually come to play an energetic role in the studies of haze pollution, and many theories have been subsequently proposed and developed. In the early stage, the environmental Kuznets curve (EKC) theory, a relatively mature theoretical analysis paradigm, was constructed by Grossman and Krueger, proposing that environmental pollution would present an inverted U-shaped trend that deteriorates first and then improves with economic growth [38]. Subsequently, a large number of theoretical and empirical studies have been carried out, but there have been constant disputes and controversy about EKC theory for a long time. At the same time, classical environmental economics theories such as like benefit-cost analysis, externality theory and system science theory are widely used to study the economic causes and the mechanisms of impact of haze pollution [1,13,19,103]. Furthermore, the complexity of the causes of haze, the spillover of pollution transmission and the ambiguity of governance boundaries mean that multiple subjects are required to participate in the process of haze governance. Environmental regulation theory, cooperative game theory and stakeholder theory are carried out as the main analytical frameworks to study the management strategies and influence paths of haze pollution control [34,45,65,100,104,105]. The evaluation and analysis of haze policy mainly rely on resource curse theory, synergy theory and benefit-cost analysis in the existing literature [17,90,91,106]. Hence, recent studies on the causes, control measures and policies in relation to haze pollution are reviewed in this section from the perspective of their theoretical bases and analytical frameworks, as presented in Table 3.

### 5.2. Empirical Methods and Quantitative Models

As is mentioned in a great amount of empirical studies, quantitative methods and models have been employed to identify causality and effect mechanisms, to measure environment governance efficiency, and to forecast and evaluate the effectiveness of related policy. Table 4 summarizes the major methods and models used in the current literature, together with the discussions on their main features and the scopes of their application. Moreover, despite significant progress, different parts of the methods in the existing studies require different improvements in order to enhance their performance. For example, many studies have focused on the spatial autocorrelation of haze pollution, spatial spillover effects and spatial correlation characteristics. According to “Tobler’s First Law of Geography”, most spatial data are spatially dependent and feature accumulation and spillover characteristics. Therefore, spatial econometric methods such as spatial regression models, geographically weighted regression models, spatial simultaneous equation models and the exploratory spatial data analysis model have been commonly used in existing researches [7,57,58]. China’s haze pollution has a significant spatial spillover effect, which is manifested as the agglomeration of high-value haze pollution areas and higher pollution-level areas, whereas low-value haze pollution areas tend to agglomerate with lower-level areas. [59]. Furthermore, when it comes to causal recognition and mechanism of the effects of economic and social factors on haze pollution, the existing research has mostly relied on the improvement and expansion of the multiple linear regression model, the KAYA identical equation, the STIRPAT model, and the evolutionary game model [32,44,45,46]. The factors studied mainly include economic development level, industrial structure, urbanization, population agglomeration, foreign direct investment, energy consumption, modes of transportation, fiscal decentralization, environmental governance, public concern and so on [47,48,49,50,51,52,53]. Furthermore, certain quantitative models such as the data envelopment analysis model, the multi-area input-output model and the computable general equilibrium model have also been employed to study the indicator system and quantitative evaluation. On the above basis, spatial economic theory, as an interdisciplinary subject of regional science, as well as new economic geography, econometrics and geographic computing science, have been widely applied in studies on haze pollution, resulting in much progress, but the imperfections and deficiencies of the existing models still require further breakthroughs, and this is regarded as one of the key directions of future research.

## 6. Conclusions and Future Research Outlook

This section may be divided by subheadings. It should provide a concise and precise description of the experimental results, their interpretation, as well as the experimental conclusions that can be drawn.

### 6.1. Major Research Findings and Limitations

Regional haze pollution, as a complicated environmental problem, as well as comprehensive academic issue, has been studied from an interdisciplinary perspective by researchers with diverse backgrounds. This study is a systematic literature review of recent studies concerned with the formation, governing strategies and policy analysis in relation to regional haze pollution. The existing literature can be divided into three major categories of studies, consisting of the basic research on formation, indicators and characteristic attributes of regional haze pollution; theoretical and empirical research on its economic and social impacts and effects; and policy research on quantitative assessment and effectiveness analysis, respectively from the perspectives of natural science as well as social science. Certain progress has been made in the research on regional haze pollution, both in the theoretical analysis frameworks, as well as empirical methods and models [107,108,109,110,111,112,113,114,115,116]. The major research findings are as follows. (1) Haze is composed of a large number of fine dry particles, accompanied by the accumulation of secondary aerosols. The formation of haze pollution is caused by the combination of special meteorological conditions, local pollutant emissions, geographic location characteristics and regional pollutant transmission. The measurement indicators of haze pollution involve a combination of concentration data from ground monitoring and the remote sensing data. (2) Haze pollution has obviously complex components and cross-regional characteristics. China’s haze pollution distribution shows significant spatial autocorrelation and spatial spillover effects, which is not only affected by economic factors, but also has a profound impact on the economic and social environment. (3) China’s system of policies on haze pollution has been gradually established and steadily promoted, and industrial development and corporate transformation are important regulatory entities, but there is a lack of restraint and regulation on residential consumption and public behaviors in current policies. Governance strategies and policy implementation need to break the boundaries of administrative jurisdictions and establish a cross-regional coordination mechanism. (4) Spatial economic theory, as an interdisciplinary subject of regional science, as well as new economic geography, econometrics and geographic computing science, have been widely applied in studies on haze pollution, but the updating and development of theoretical foundations and research methods are slow, with few pioneering results and further breakthroughs. However, many deficiencies and challenges remain in the current literature, as follows. (1) The concept of haze pollution and its measurement indicators have not been authoritatively defined, as precise concepts and definitions are the basis of this definition. In practice, it is difficult to accurately identify haze pollution and its differences in different regions, causing a lack of pertinence and effectiveness in the formulation of smog control policies. (2) Based on the characteristics of the temporal and spatial evolution of haze pollution, existing studies pay great attention to the influence of the spatial spillover effect, but ignore the time lag effect and the time accumulation effect of the formation and evolution process of haze pollution. (3) The current empirical models and analysis methods are traditional and monotonous, with few pioneering findings and theoretical breakthroughs. Relevant studies are mostly carried out from the perspective of the spatial and empirical level, lacking causal recognition and theoretical derivation. This field of study has long relied on spatial econometric regression models, geographically weighted regression models and spatial simultaneous equation models. (4) Research on cross-regional pollution control strategies mainly uses cooperative game methods from the perspective of maximizing regional economic benefits, but lacks research on high-quality economic, social and environmental development paths from the perspectives of endogenous growth theory and multi-regional integration. Recent policy formulations and implementations lack pertinence and continuity, and policy research lacks effective evaluation and effectiveness analysis. (5) The current governance strategies mainly rely on pollutant control and emission reduction effects, led by single government and administrative regulations, and the existing studies pay little attention to the efficiency and quality of haze control and the synergistic effect of climate change and carbon emissions from a carbon-neutral perspective. Certain problems remain in relation to policy research on the coordinated control and synergistic governance of multiple air pollutants, carbon emissions and climate change targets.

### 6.2. Future Direction and Discussions

Based on the existing problems as identified in previous literature reviews [117,118,119,120,121,122], we believe that future research can be complemented and expanded in the following aspects. (1) It is imperative to construct an indicator system and authoritative definitions that are in accordance with China’s actual conditions from the perspectives of natural science and economic management, by which it is convenient to accurately grasp the composition, attribute and governance stage of different types of haze pollution in different areas, to provide a basis for the effective formulation and implementation of policies. (2) It is critical to study the win-win pathways and synergistic effects of urbanization in promoting economic growth and haze governance. In the process of promoting the construction of urban agglomerations and metropolitan areas, it is necessary to explore urban agglomeration construction models and coordination mechanisms under the constraints of haze pollution and to realize the goal of “decoupling” of urbanization. (3) It is urgent to explore the coordinated mechanisms of emissions reductions of multiple air pollutants, and form a coordinated development model for haze pollutant control and climate change targets. China’s urban air environment, which has long centered on haze pollution, has the characteristics of diversified pollutants, with serious and extensive regionalization. At present, ozone has become the most serious air pollutant in the summer, so it is also necessary to explore the collaborative governance relationship between multiple air pollutants to reduce the cost of governance and to achieve the simultaneous target of improving haze quality and addressing climate change goals. (4) It is necessary to explore the incentive policies and pathways for haze pollution control involving multiple stakeholders, such as environmental regulation, market incentives and social organization participation, and to focus on economic incentive policies and implementation pathways from the perspective of “Pigovian tax” theory and Coase’s theory of property. (5) It is necessary to further explore new theories, new methods and new pathways for social science research, such as the economic and management issues related to haze pollution, on the basis of existing traditional theories and methods such as the spatial econometrics paradigm, to explore evolutionary game models, input-to-production analysis, social network analysis, data mining and big data analysis, as well as new structural economic theory, and decoupling theory in conducting research on the identification of the cause-and-effect mechanisms of haze pollution and the evaluation of government policies.

## Figures and Tables

**Table 1:** Comparative analysis and evaluation of measurement indicator systems for haze pollution.

Category	Indicator Content	Introduction	Advantages	Limitations	Applicable Scope
Emission statistics	industrial pollutant emissions	independent statistics on corporate emissions	better continuity and availability	the coverage is not comprehensive, and the statistical caliber varies greatly	industry level
Surface pollutant monitoring	particulate matter	environmental monitoring data on the concentration of ground pollutants	better effectiveness and timeliness	affected by the distribution of monitoring points, human operation error interference	national/ provincial
Satellite remote sensing monitoring	optical depth/ concentration	satellite remote sensing monitoring and pollutants data conversion	wide monitoring range, high reliability of full-caliber statistical data less statistical error	affected by meteorological conditions and atmospheric environmental conditions; the identification of subdivided pollutants is low	regional/ global
Comprehensive air index	air quality index/air pollution index	comprehensive data weighted based on air quality standards and the impact of pollutants	high-frequency data, the same statistical caliber	affected by air quality standards and personal error	national/ city level
Others	days of up-to-standard weather	statistical data for the overall judgment of annual air quality	comprehensively reflect the city’s annual air quality status	difficult to unify statistical standards	city level
	home indoor survey	household survey data based on indoor air pollution	truly and effectively reflect the situation of indoor air pollutants	affected by subjective emotions, interference by personal error	city level

**Table 2:** Representative documents and statistics on China’s policies in relation to haze pollution prevention and control.

No.	Policy Name	Release Year	Release Department
1	Law of the People’s Republic of China on the prevention and control of atmospheric pollution	1988, 2016, 2018	The National People’s Congress
2	Guidance on promoting the joint prevention and control of air pollution and improving regional air quality	2010	The State Council
3	The air pollution prevention and control plan	2013	The State Council
4	The clean air action plan in Shanghai (2013–2017)	2013	Shanghai Municipality
5	Municipal air pollution prevention and control regulations	2014	Beijing Municipality
6	Measures for the prevention and control of haze pollution in Sichuan Province	2015	Sichuan Province
7	Notice on the implementation plan of full-compliance emissions for industrial pollution sources	2016	Ministry of Ecology and Environment
8	Work program for air pollution prevention and control around Beijing–Tianjin–Hebei and the surrounding urban agglomeration	2017	Cooperative group of air pollution prevention and control
9	The three-year action plan for winning the “blue sky defense war”	2018	The State Council
10	The comprehensive control action plan for air pollution in autumn and winter around the Beijing–Tianjin–Hebei urban agglomeration	2018	Ministry of Ecology and Environment
11	The linkage support work plan for winning the “blue sky defense war”	2020	Sichuan Province and Chongqing Municipality

**Table 3:** Summary of mainstream theoretical bases of studies on haze pollution control.

Category of Theoretical Basis		Core Point of Views	Main Features	Applicable Scope
Environmental Regulation theory	Porter Hypothesis	Environmental regulations can improve the production efficiency and competitiveness of enterprises with innovation compensation effects, and achieve a win-win situation for environmental protection and economic growth.	The strong Porter hypothesis, weak Porter hypothesis, narrow Porter hypothesis	Enterprise environmental technology innovation capability
	Race-to-the-Bottom Hypothesis	Developing countries or regions with strict environmental regulations tend to lower environmental regulatory standards to attract the participation and entry of external enterprises to stimulate economic growth and enhance industrial competitiveness	Race-to-the-bottom or race-to-the top	The impact of industrial structure changes
	Pollution Haven Hypothesis	Pollution-intensive multinational companies move to countries with loose environmental regulations with the large-scale transfer of pollutants to circumvent the domestic strict environmental regulations.	Phenomenon of pollution refuge or pollution paradise	Foreign direct investment analysis
Resource Curse theory		Abundant natural resources may be a curse of economic development, rather than an advantage. Most countries with rich natural resources grow more slowly than those with scarce resources.	Dutch disease phenomenon	Rich in natural resources or endowments
Stakeholder theory		Stakeholders influence the company’s long-term strategic goals, and it is necessary to clearly incorporate the interests of stakeholders into the company’s strategic decision-making in order to efficiently allocate and manage a lack of resources.	Enterprise dependence, strategic management and ownership distribution	Multi-subject participation and coordination in environmental governance
Benefit-cost analysis		The main body engaged in economic activities tends to acquire profit maximization with the maximum benefit and the minimum cost. The reason people want to carry out cost-benefit analyses is to obtain the maximum benefit with the least input.	Pursue the maximization of utility; the characteristics are self-interest, economy and calculation	Consider the gains and losses of specific economic actions in terms of economic value
Synergy theory		Taking the principle of self-organization as the core, emphasizing that the internal subsystems of the system form a certain structure and function according to certain rules, and mainly study the issue of multi-center subjects participating in the governance process.	System environment, collaborative process and synergistic effect	Diversified governance bodies, self-organization and common rules
Environmental Kuznets Curve		There is a certain regularity hypothesis relationship between economic growth and environmental quality, showing an inverted U-shaped change in the long run.	Scale effect, structure effect, technical effect	The long-term impact of the environment
System Science theory		Only when the various elements in the system reach a state of harmonious coexistence, can the system be called a coordinated development system.	The relationship between various elements in the system and its dynamic changes	Energy–economy–environment system
Externality theory		Externalities exist when a consumption or production activity has an indirect effect on other consumption or production activities that is not reflected in the market price	External cost, external effect or spillover effect	Perfectly competitive economic activity
Cooperative game theory		The interests of both parties in the game have increased, or at least the interests of one side have increased, while the interests of the other side are not harmed, so the interests of the entire society have increased	Divided into cooperative game and non-cooperative game theory, according to whether a binding agreement can be reached	Problem of income distribution

**Table 4:** Summary of empirical analysis and quantitative methods of haze pollution control.

Study Method or Model	Introduction	Main Features	Applicable Scope
Multiple linear regression model	A dependent variable is affected by multiple independent variables, and the relationship between each independent variable and the dependent variable is linear.	Correlation analysis is used to measure the strength of the association between several variables.	Identifies the correlation and causality between multiple variables.
Spatial econometric regression model	This method is mainly used to study problems with spatial dependence, adding spatial interaction effects on the basis of time and individual effects, including the spatial lag model, the spatial error model and the spatial Durbin model, which are widely used as optimal models.	A spatial autocorrelation test needs to be performed on spatial data; the spatial weight value includes three methods of adjacency, geographic distance and economic distance, and the appropriate weight value method needs to be selected.	It is suitable for panel data and is mostly used to analyze the direct and indirect effects of variables.
Geographically weighted regression model	This method incorporates the spatial correlation into the regression model, uses the relevant information from neighboring regions to estimate the local regression parameters, and realizes that the coefficients of the regression model in different regions change with spatial changes.	It is a cross-sectional data model; the value of the spatial weight includes the distance threshold method, the inverse distance method, the Gaussian function method, etc.; through the regression of each area one by one, the parameter matrix of all areas to be estimated is obtained.	The time series of the sample data is short and the cross-sections vary greatly.
Spatial simultaneous equation model	This method combines multiple regression equations to explore the impact and spatial effects of various factors on the research object. It is usually estimated using the two-stage or three-stage least squares method.	Reflecting the interactive influence and spatial effects among various variables, the method of selecting spatial weights is similar to that of spatial measurement models.	The interaction between spatial effects and variables. Response mechanism.
Evolutionary game model	This method combines game theory analysis and dynamic evolution process analysis, which emphasizes a dynamic equilibrium.	Analyzing the factors affecting the formation of social habits, norms, institutions or institutions and explain their formation process	Assumes that participants are completely rational and have consistent preferences.
Data envelopment analysis model	Quantitative analysis method, used to evaluate the relative effectiveness of comparable units of the same type.	A method of linear programming with multiple input indicators and multiple output indicators.	Affected by the selection of cost indicators and benefit indicators.
Computable general equilibrium model	This method can be used to analyze the economic impact of many variables and address regional issues within and between countries through multi-regional analysis.	It has the characteristics of computability, generality and balance	Real economic data are used as an input and the equilibrium solution is calculated.
Multi-area input-output model	This method can comprehensively analyze the quantitative dependence between inputs and outputs in economic activities.	It is composed of two parts: the input–output table and the mathematical equations established according to the balance relationship of the input–output table.	Analyzing and examining the quantitative dependence between product production and consumption in various sectors of the national economy
Spatial auto-correlation analysis	This method is used to measure the spatial distribution characteristics of physical or ecological variables and their influence on the field.	Global autocorrelation coefficient and local autocorrelation coefficient.	Correlation analysis can detect whether there is a correlation between changes in two phenomena (statistics).

## Data Availability

No new data were created or analyzed in this study. Data sharing is not applicable to this article.

## Abbreviations

Summary of abbreviations in the text.

**Abbrev.**	**Explanation**
PM2.5	Fine particulate matter
PM10	Inhalable particulate matter
AQI	Air quality index
API	Air pollution index
SO2	Sulphur dioxide
CO2	Carbon dioxide
NOx	Nitrogen oxides
O3	Ozone
VOCs	Volatile organic compounds
EKC	Environmental Kuznets curve
STIRPAT	Stochastic impacts by regression on population, affluence and technology
GMM	Gaussian mixed model
SAR	Spatial auto regression model
SLM	Spatial lag model
SDM	Spatial Dubin model
SEM	Spatial error model
GDP	Cross domestic product
GIS	Geographic information system
